# Global patterns of modularity and narrow host use in fish-parasitic copepods (Crustacea)

**DOI:** 10.3897/BDJ.13.e163693

**Published:** 2025-09-15

**Authors:** Francisco Neptali Morales-Serna

**Affiliations:** 1 Instituto de Ciencias del Mar y Limnología, Universidad Nacional Autónoma de México, Mazatlán, Mexico Instituto de Ciencias del Mar y Limnología, Universidad Nacional Autónoma de México Mazatlán Mexico

**Keywords:** macroecology, parasites, host-specificity, network, marine, freshwater

## Abstract

Parasitic copepods are amongst the most diverse and ecologically significant parasites of fish, yet their macroecological patterns remain poorly understood. In this study, a comprehensive dataset of fish–copepod associations was obtained and curated from the World of Copepods database. After taxonomic validation and filtering, the dataset included 2296 copepod species parasitising 2690 fish species from both marine and freshwater ecosystems. Host use was quantified at species, genus and family levels, revealing a predominance of specialists in both environments. In marine ecosystems, *Caligus elongatus*, a species of concern for aquaculture, was identified as the most generalist copepod. In freshwater ecosystems, *Lernaea cyprinacea*, considered invasive in several regions, was the most generalist species. Bipartite network analysis revealed high modularity and low nestedness in both habitats, suggesting compartmentalised host–parasite structure. Robustness simulations indicated that the loss of key fish hosts, especially several threatened elasmobranchs in marine ecosystems, could disproportionately impact parasite diversity. This study highlights the importance of host identity in shaping parasitic copepod assemblages and provides a global baseline to assess ecological vulnerability in host–parasite networks.

## Introduction

Parasitic copepods exhibit considerable diversity in terms of morphology and species richness, with over 4000 species described from all major phyla of marine animals, ranging from sponges to mammals and over 300 species reported from freshwater hosts, primarily fishes, but also molluscs (Ho 2001, Boxshall and Defaye 2008, Boxshall and Halsey 2019, Boxshall and Hayes 2019). Despite their broad host range, parasitic copepods are especially common and abundant on fishes, where they occupy a variety of microniches including the skin, gills, fins and body cavities (Kabata 1979, Boxshall and Halsey 2019). Their diversity and ubiquity make them important components of aquatic parasite communities and, in some cases, significant agents of disease in farmed fish, such as *Lepeophtheirus salmonis* and *Caligus rogercresseyi*, which pose serious challenges in salmon aquaculture (Johnson et al. 2004, Costello 2009).

Large, taxonomically standardised datasets provide valuable opportunities to uncover macroecological patterns in host–parasite associations that are often overlooked in smaller or regional studies (Poulin 2007, Dallas et al. 2018, Krasnov et al. 2022). By analysing broad-scale records, researchers can explore general trends in host use, parasite diversity and the ecological and evolutionary processes that shape parasite assemblages. These approaches have been successfully applied to helminth parasites of fishes to evaluate host specificity, network modularity and robustness (Strona et al. 2013, Braga et al. 2014, Bellay et al. 2015, Dallas and Cornelius 2015, Llopis-Belenguer et al. 2020), but parasitic copepods remain largely absent from such global analyses despite their diversity and ecological importance.

This study explores host–parasite interactions involving fish-parasitising copepods using a curated dataset derived from the World of Copepods database (Walter and Boxshall 2025). Host use was described at the species, genus and family levels for both marine and freshwater systems, with particular attention to the most generalist and specialist copepod species. Additionally, host–parasite networks were characterised using metrics of modularity, nestedness and robustness to host loss. By applying a standardised taxonomic workflow, this study documents patterns of copepod host associations and network structure across distinct aquatic environments. These findings have implications for parasite biodiversity, disease ecology and conservation, particularly in the context of declining fish populations and increasing pressures on aquatic ecosystems.

## Material and methods

**Data source and curation**

The dataset analysed in this study was obtained from the World of Copepods database (Walter and Boxshall 2025), hosted by the World Register of Marine Species (WoRMS; www.marinespecies.org). Since the entire dataset is not publicly downloadable in a single file through the WoRMS platform, the complete set of records, updated to May 2025, was kindly provided directly by Chad Walter (Smithsonian Institution), the chief taxonomic editor of the database. The original records included host–parasite associations involving parasitic copepods and a wide range of vertebrate and invertebrate hosts. To restrict the analysis to fish–copepod interactions, the dataset was filtered to retain only records involving host species belonging to the superclass Pisces, including both teleosts and elasmobranchs. Taxonomic names of fish hosts were validated and standardised using the taxonomic backbone from FishBase, implemented through the R package rfishbase (Boettiger et al. 2012). Habitat information for each host species was retrieved using the species() function in rfishbase. Based on the occurrence flags provided by FishBase, fish species were classified into two broad categories: marine (if they occurred in brackish or saltwater environments) and freshwater (if restricted to freshwater environments). All data curation, integration and filtering procedures were conducted in Python 3.11, using the libraries pandas (McKinney 2010) and numpy (Harris et al. 2020). In particular, the rfishbase package was run in R version 4.3 (R Core Team 2023) to retrieve habitat information for each fish species. Only unique host–parasite species combinations were retained for downstream ecological and network analyses.

**Host use**

Host use was assessed at three hierarchical taxonomic levels: fish species, genus and family. Analyses were based on a dataset of unique host–parasite associations, where each record corresponded to a single copepod species parasitising a single fish species. For each copepod species, the number of distinct host taxa at each level (species, genus and family) was recorded. Genus and family affiliations were assigned, based on the taxonomic hierarchy in FishBase and derived directly from the species-level interactions, ensuring that no duplicate or overlapping records were introduced. Copepods associated with only one host species, genus or family were classified as specialists at the corresponding level. To minimise potential sampling bias in family-level assessments, a subset of specialists was further filtered to include only those species with at least three independent host interaction records. The most generalist copepods were identified as those associated with the highest number of host families.

**Network Analyses**

Bipartite ecological networks were constructed to represent host–parasite associations between parasitic copepods and their fish hosts. Each network was built using undirected and unweighted links, where edges connected copepod species to their respective fish host taxa. For most analyses, networks were constructed at the level of fish families to facilitate clearer visualisation and reduce sampling biases, as family-level associations are less sensitive to incomplete host records and are considered a robust proxy for ecological specialisation (Poulin 2007).

Key network metrics were calculated to characterise the structure and complexity of the host–parasite networks. Connectance was defined as the proportion of realised links out of all possible links, indicating overall network density. Modularity (Q) was measured using the Louvain method, which detects communities (modules) of species that interact more frequently with each other than with species outside their module. Modularity values range from 0 to 1, with higher values indicating stronger compartmentalisation, which may enhance network stability and resistance to perturbation (Olesen et al. 2007, Dormann and Strauss 2014). Nestedness was assessed using the Nestedness metric, based on Overlap and Decreasing Fill (NODF), which evaluates whether specialists interact with subsets of hosts used by generalists (Almeida-Neto et al. 2008). NODF values range from 0 to 100, with higher values indicating a more nested structure. High nestedness is thought to confer robustness to species loss (Bascompte et al. 2003).

To assess network robustness, sequential removal of fish species from the network was simulated and the fraction of copepod species that remained connected to at least one host was monitored. Two removal strategies were implemented: (1) random extinction, in which host species were removed in a random order, repeated over 100 simulation runs to account for stochastic variation; and (2) targeted extinction, in which hosts were removed in descending order of their degree (i.e. number of parasite associations). Robustness was inferred from the shape of the resulting curves, with steeper declines indicating greater vulnerability (Dunne et al. 2002). Simulated species removal has been widely used as a proxy to evaluate ecological network robustness, providing insights into structural redundancy and susceptibility to species loss (Memmott et al. 2004, Dallas and Cornelius 2015). The comparison between random and targeted scenarios allows assessment of whether the network's integrity depends disproportionately on highly connected host species.

All analyses were conducted in Python 3.11 using the libraries pandas for data handling, networkx (Hagberg et al. 2008) for network construction and metric calculation, community-louvain (Blondel et al. 2008) for community detection using the Louvain method and matplotlib (Hunter 2007) for network visualisation.

## Results

### Dataset summary

The curated dataset comprises a total of 2296 species of parasitic copepods from 333 genera and 35 families, recorded infecting 2690 fish species, belonging to 1346 genera and 325 families. These records represent 8037 unique host–parasite species associations. When disaggregated by habitat, the marine subset includes 2193 copepod species (from 320 genera and 32 families) infecting 2287 fish species (from 1202 genera and 310 families), totalling 7106 unique interactions. The freshwater subset contains 273 copepod species (from 82 genera and 19 families) parasitising 403 fish species (from 277 genera and 109 families), with 931 unique associations. Additionally, 101 fish species lacked habitat information and were excluded from habitat-specific analyses. Some taxa occur in both habitats, so the subsets are not mutually exclusive.

### Host use in marine parasitic copepods

**Figure 1.**
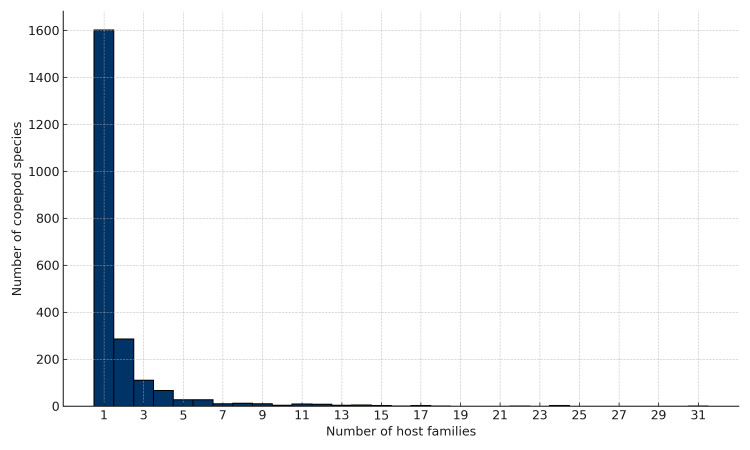
Distribution of host family range amongst marine parasitic copepod species.

Host use was evaluated at three taxonomic levels: fish species, genus and family, for 2193 marine parasitic copepod species. A total of 1104 species (50%) were classified as specialists at the species level, parasitising a single fish species. At the genus level, 1327 copepod species (61%) were associated with only one fish genus. Family-level specificity was even more pronounced, with 1601 species (73%) restricted to a single host family, indicating a strong overall tendency towards specialisation. The distribution of host family ranges was highly skewed, with most copepods infecting only one host family and relatively few exhibiting broader associations (Fig. 1).

**Figure 2.**
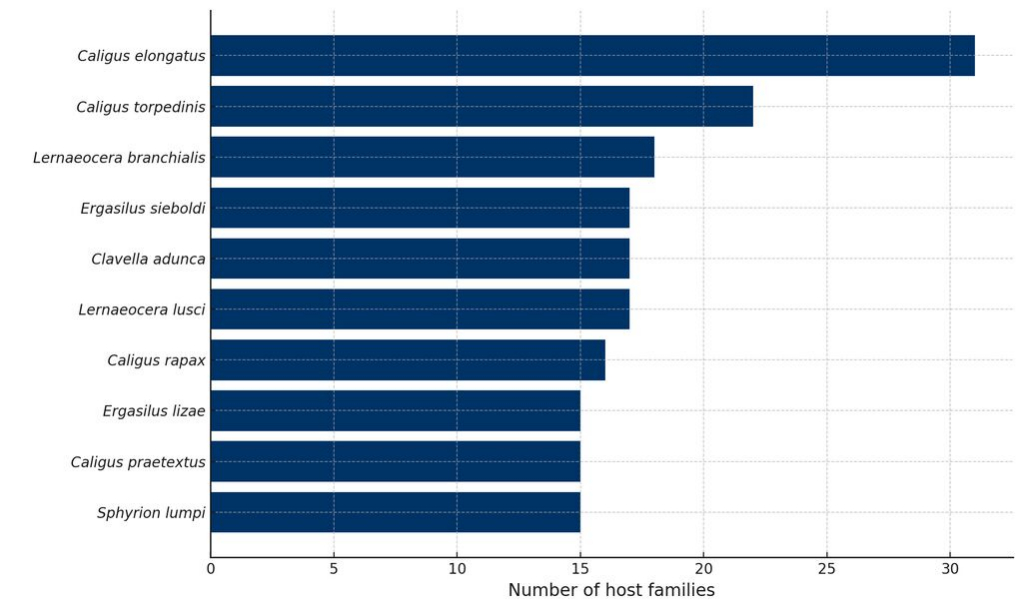
Top 10 marine parasitic copepod species with the broadest host family range.

To identify the most specialist copepods, while reducing potential sampling bias, only species with at least three host records were considered. Amongst these, ten marine copepod species were identified as the most specialised, each being associated with a single fish family. These included *Parabrachiella robusta* (30 records), *Procolobomatus kyphosus* (18), *Colobomatus embiotocae* (15), *Chondracanthus triventricosus* (15), *Hatschekia pygmaea* (13), *Clavellotis sargi* (12), *Caligus asymmetricus* (11), *Colobomatus belizensis* (10), *Clavellotis fallax* (10) and *Lernanthropus belones* (10). These species exemplify strong host fidelity despite being recorded multiple times, suggesting true ecological specialisation rather than sampling artefacts. Conversely, the top ten generalist copepod species were each associated with hosts from ten or more fish families. Notably, *Caligus elongatus* emerged as the most generalist species, infecting fishes from 31 distinct families (Fig. 2).

### Marine network structure

**Figure 3.**
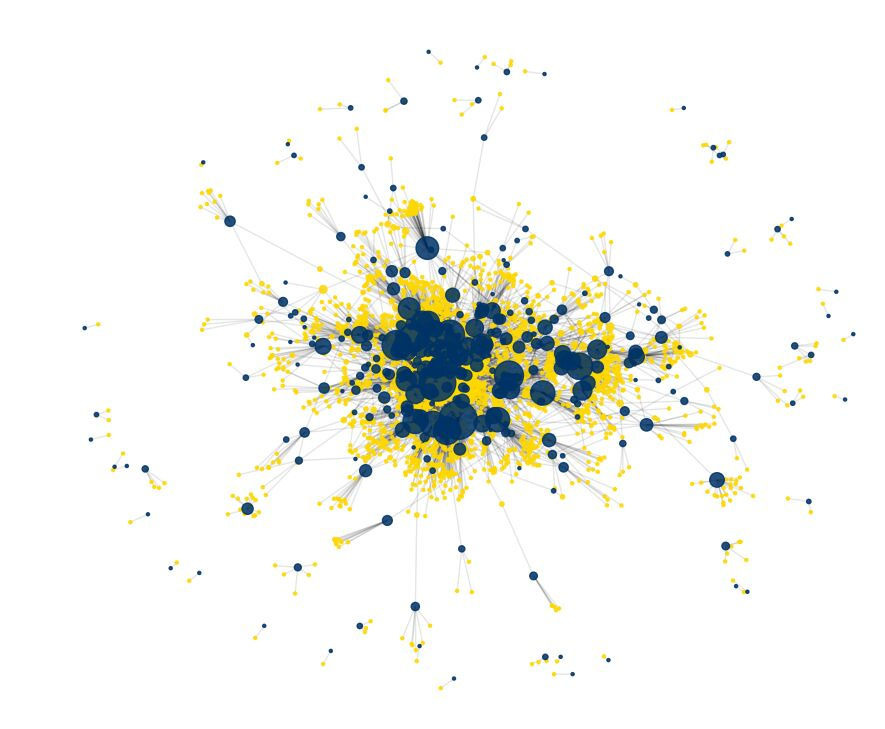
Bipartite network showing interactions between parasitic copepods (yellow nodes) and marine fish families (dark blue nodes). Node size is scaled to represent node degree (number of connections) and the spring layout emphasises clusters and interaction density.

The marine bipartite network, built at the level of host families and parasite species, comprised 310 fish families and 2193 species of parasitic copepods, linked by 3936 unique interactions (Fig. 3). This number is lower than the total species-level associations in the marine dataset (n = 7106) because fish hosts were aggregated at the family level for network construction, collapsing multiple copepod–fish species interactions into a single link per family. The network showed low connectance (0.016). Modularity (Q = 0.72) was high, reflecting a strongly compartmentalised architecture, with distinct modules grouping subsets of host families and their associated copepods. In contrast, the network showed low nestedness (NODF = 9.6), suggesting that copepod species tend not to infect subsets of host families used by generalist species.

**Figure 4.**
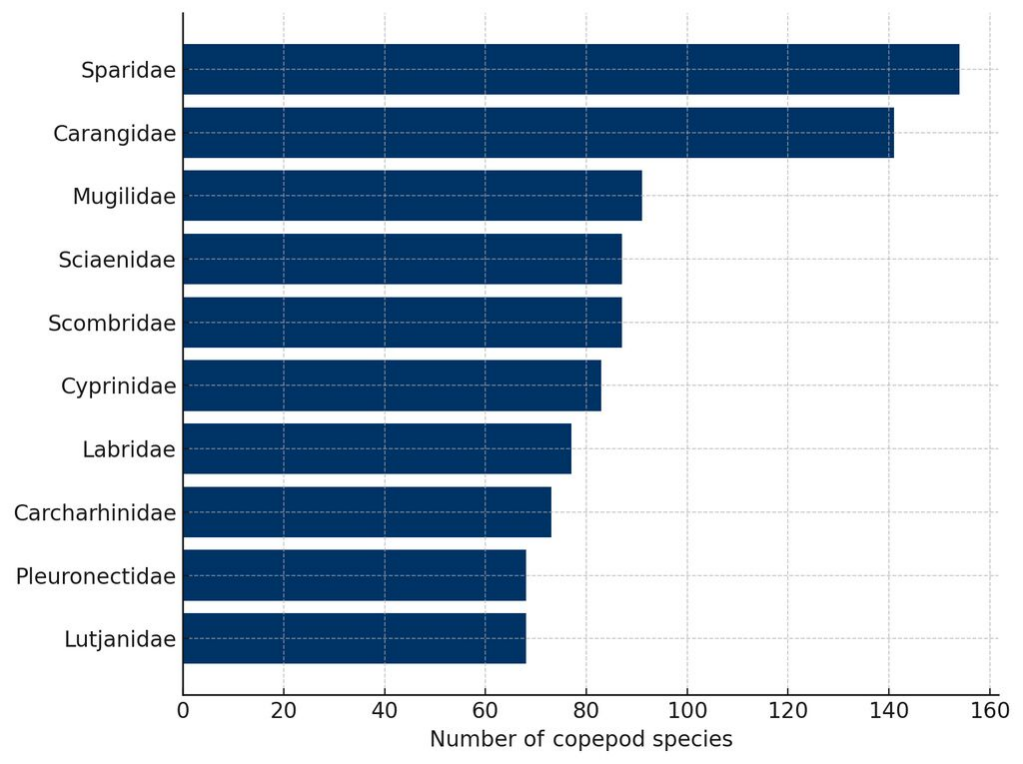
Top ten marine fish families with the highest number of associated copepod species. These families act as highly connected hubs within the parasite network.

The marine fish–parasite network revealed strong heterogeneity in host connectivity. The fish family Sparidae was the most connected, hosting 154 copepod species, followed by Carangidae (141 species) and Mugilidae (91 species). Other highly connected families included Sciaenidae, Scombridae and Cyprinidae, each associated with more than 80 copepod species (Fig. 4). These families likely represent important hubs in the network structure.

**Figure 5.**
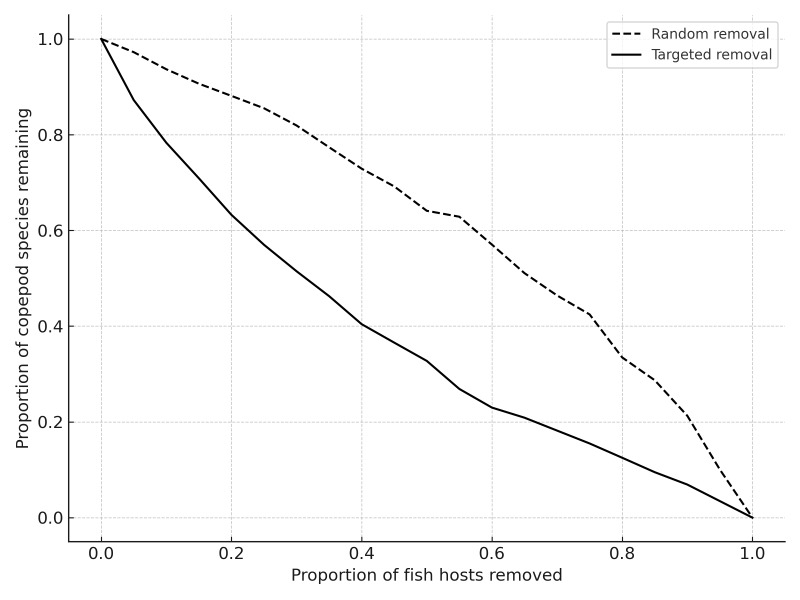
Robustness of the marine host–parasite network to species loss. The graph shows the proportion of copepod species that remain connected to at least one fish host after sequential removal of fish species. Dashed line represents random extinction; solid line represents targeted removal of the most connected fish species first.

Simulated removal of fish species from the marine host–parasite network revealed differences in copepod persistence under random versus targeted extinction scenarios. The fraction of copepod species that remained connected to at least one host declined more rapidly when the most connected fish species were removed first, compared to random removal. This indicates that the network is more vulnerable to the loss of highly connected hosts (Fig. 5). The most connected fish species, based on the number of associated copepod species, were *Mugil cephalus* (59), *Prionace glauca* (29), *Oncorhynchus mykiss* (27), *Katsuwonus pelamis* (25), *Isurus oxyrinchus* (25), *Squalus acanthias* (24), *Sphyrna mokarran* (24), *Gadus macrocephalus* (22), *Dicentrarchus labrax* (21) and *Rhizoprionodon acutus* (21).

### Host use in freshwater parasitic copepods

**Figure 6.**
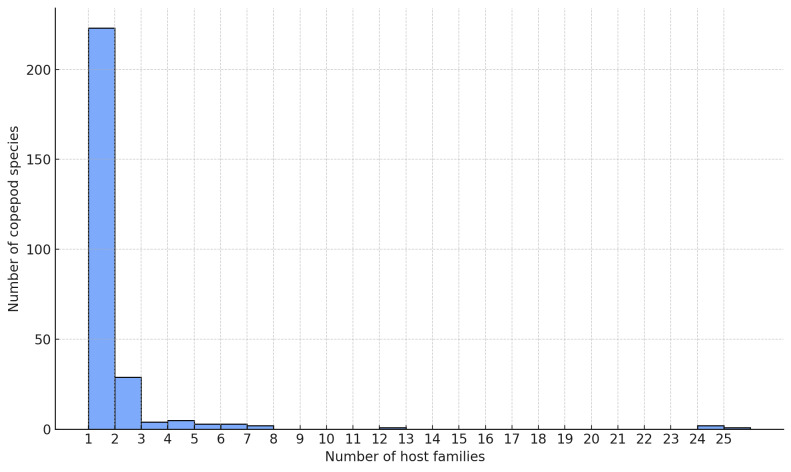
Distribution of host family range amongst freshwater parasitic copepod species.

Host use was evaluated at three taxonomic levels (species, genus and family) for the 273 freshwater parasitic copepod species. At the species level, 183 copepods (67%) parasitised a single fish species and 260 (90%) infected three or fewer species. At the genus level, 197 species (72%) were associated with a single host genus. At the family level, the distribution of host range was highly skewed, with most species parasitising hosts from a single family (Fig. 6). Specifically, 223 copepod species (82%) were classified as specialists, being associated with only one fish family. These results indicate a consistent pattern of specialisation in freshwater systems, which is particularly robust at the family level.

**Figure 7.**
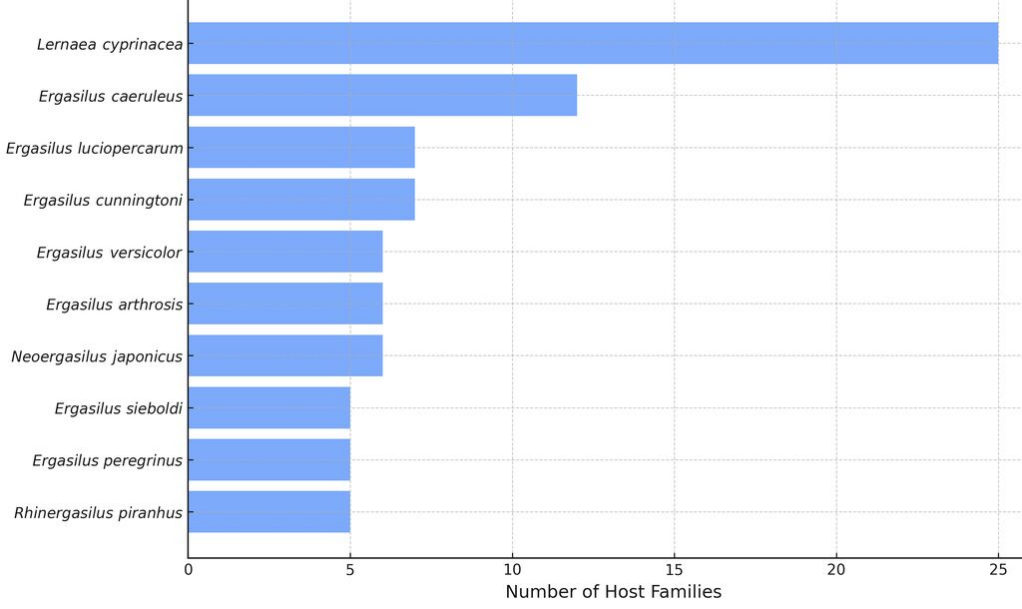
Top 10 freshwater parasitic copepod species with the broadest host family range.

At the fish family level, ten copepod species were identified as strong specialists, being restricted to a single host family and supported by multiple interaction records. These included *Lamproglena monodi* (19 records), *Tracheliastes polycolpus* (9), *Ergasilus megacheir* (8), *Ergasilus parasarsi* (7), *Acusicola tenax* (6), *Lamproglena compacta* (4), *Ergasilus parvus* (4), *Achtheres micropteri* (4), *Ergasilus caparti* (3) and *Ergasilus turkanae* (3). These species represent robust examples of host specialisation in freshwater systems, with repeated observations reinforcing their consistent association with a narrow taxonomic range of hosts. In contrast, *Lernaea cyprinacea* was the most generalist freshwater copepod species, being associated with fish from 25 different families. The remaining top nine generalist species all belonged to the family Ergasilidae (Fig. 7).

### Freshwater network structure

**Figure 8.**
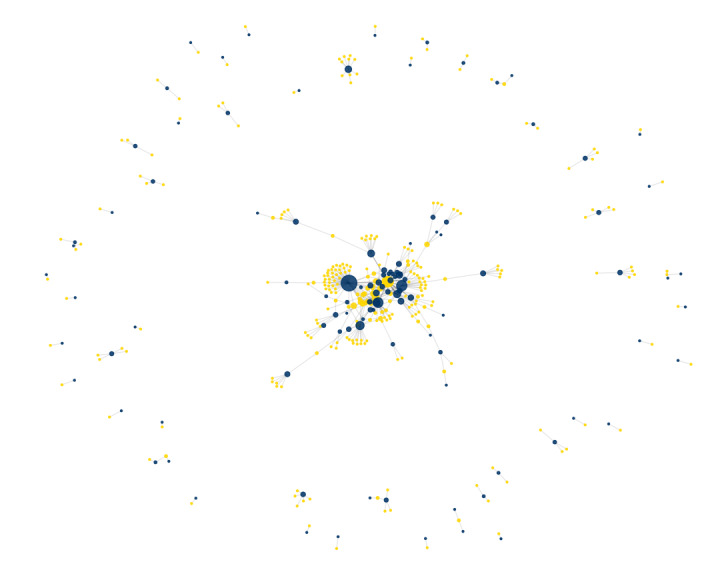
Bipartite network showing interactions between parasitic copepods (yellow nodes) and freshwater fish families (dark blue nodes). Node size is scaled to represent node degree (number of connections) and the spring layout emphasises clusters and interaction density.

The freshwater host–parasite network comprised 109 fish families and 273 copepod species, forming 390 unique host–parasite associations (Fig. 8). The network showed low connectance (0.015), indicating a sparse, but non-random structure. Community detection analysis revealed 117 discrete modules and the modularity index was high (Q = 0.7943), suggesting that copepod species tend to be compartmentalised by fish lineages. Nestedness was low (NODF = 5.66), consistent with the strong modular structure and low host overlap amongst generalists.

**Figure 9.**
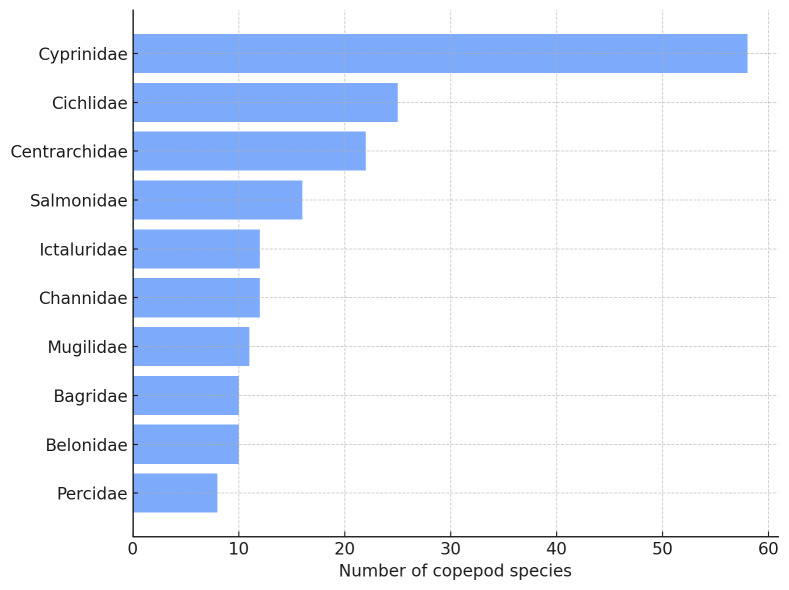
Top 10 freshwater fish host families with the highest number of associated copepod species. These families represent highly connected hubs in the freshwater host–parasite network.

The ten most connected fish families in the freshwater network were identified as key ecological hubs, each supporting between 8 and 58 copepod species (Fig. 9). These included families such as Cyprinidae, Cichlidae and Centrarchidae, which hosted the highest parasite diversity and may play a central role in maintaining network stability.

**Figure 10.**
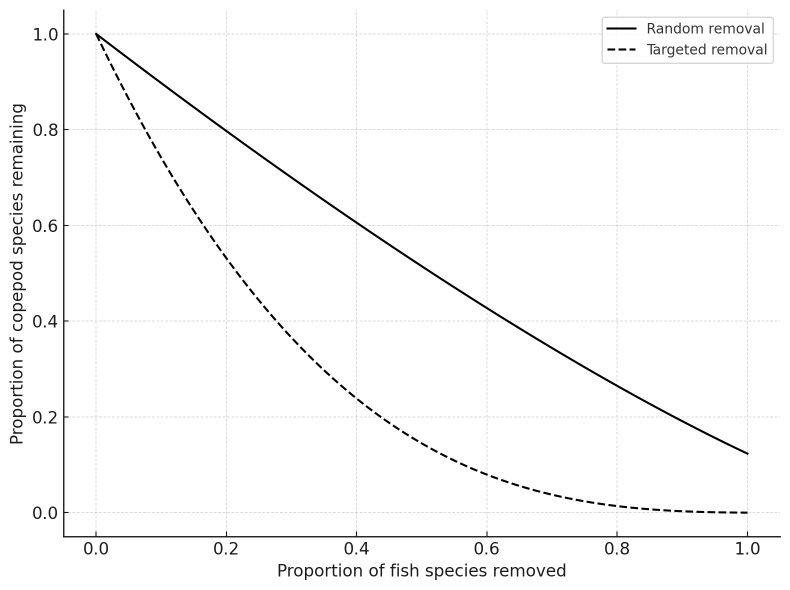
Robustness of the freshwater host–parasite network to species loss. The graph shows the proportion of copepod species that remain connected to at least one fish host after sequential removal of fish species. Solid line represents random extinction; dashed line represents targeted removal of the most connected fish species first.

Simulated removal of host species revealed that the freshwater network is more vulnerable to targeted host loss than to random extinction (Fig. 10). The number of copepod species that remained connected declined more rapidly when the most connected fish hosts were removed first. The ten freshwater fish species with the highest number of copepod associations were identified to assess their role in supporting parasite diversity and contributing to network robustness. The most highly connected hosts included *Micropterus salmoides* (14 copepod species), *Micropterus dolomieu* (12), *Ambloplites rupestris* (11), *Lepomis macrochirus* (11), *Lepomis gulosus* (10), *Barbus barbus* (9), *Ameiurus nebulosus* (9), *Channa argus* (9), *Coregonus johannae* (9) and *Clarias gariepinus* (8). These species represent critical nodes in the freshwater host–parasite network, as their removal could disproportionately impact parasite persistence and overall network stability. Several of these hosts belong to the Centrarchidae and Cyprinidae families, which were also amongst the most connected fish families in the network.

## Discussion

This study presents a global-scale analysis of host use and network structure in parasitic copepods infecting marine and freshwater fishes. Although formal metrics of host specificity were not applied, the multi-level approach (species, genus, family) offers valuable insights into host use breadth and specialisation trends. Given the challenges of measuring specificity at broad spatial scales, such as uneven sampling and taxonomic resolution (Poulin and Mouillot 2003, Wells and Clark 2019), family-level patterns provide a practical proxy, reducing bias and allowing integration with network analyses. However, it is important to acknowledge that global patterns may also be influenced by the uneven geographic distribution of sampling and research effort, which remains a limitation in parasite macroecology.

### Host use patterns

A clear tendency towards host specialisation was observed in both marine and freshwater parasitic copepods, particularly at the fish family level. This result aligns with previous findings in other parasite taxa (Poulin and Mouillot 2003, Nagel and Grutter 2007) and supports the notion that host family is a conservative and ecologically meaningful proxy for host specificity, especially when data are limited.

The evolutionary trade-offs between specialisation and generalism in parasites remain a central question in ecology. While specialisation could theoretically increase extinction risk due to dependence on narrow resources, evidence suggests that specialist parasites mitigate this risk by associating with hosts exhibiting low vulnerability traits, such as broad geographic distributions and high local abundance (Strona et al. 2013). In such cases, host stability may promote not only persistence, but also the evolution of life-history traits adapted to predictable environments. For example, Doherty et al. (2022) demonstrated that specialist fish-parasitic copepods in stable environments invest in fewer, larger, energy-rich eggs, favouring offspring quality over quantity. Although reproductive strategy and host specificity represent different ecological axes, this pattern suggests that ecological stability in host use may influence broader aspects of parasite biology, including reproductive investment.

Despite the predominance of specialists, several copepod species exhibited notably broad host ranges. In the marine dataset, *Caligus elongatus* emerged as the most generalist species, parasitising hosts from 31 fish families. This copepod is widely distributed in temperate waters and frequently associated with commercially important fish species, such as Atlantic cod (*Gadus morhua*), haddock (*Melanogrammus aeglefinus*) and Atlantic salmon (*Salmo salar*) (Johnson et al. 2004). In freshwater systems, *Lernaea cyprinacea* showed a similarly broad host range across 25 fish families. Although primarily freshwater, *L. cyprinacea* has been recorded in brackish environments and has spread widely due to aquaculture and the ornamental fish trade (Plaul et al. 2010, Avenant-Oldewage 2012). Another generalist, *Neoergasilus japonicus*, was found on hosts from seven families and is considered invasive in North America and Europe (Suárez-Morales et al. 2010, Ondračková et al. 2019). These examples illustrate how generalist copepods can facilitate host switching and geographic expansion, with implications for fish health in both wild and managed systems.

### Network structure and robustness

The marine and freshwater host–parasite networks analysed in this study exhibited significant modularity, indicating that species tend to interact within distinct subgroups or compartments. This pattern is consistent with the observed tendency towards host specialisation, particularly at higher taxonomic levels. In contrast, both networks displayed relatively low levels of nestedness. Similar patterns have been reported in previous studies, such as Bellay et al. (2015), who documented high modularity and low nestedness in a Neotropical fish–parasite network. While modularity and nestedness are often considered negatively correlated, some evidence suggests that they may also co-occur, depending on network structure and ecological context (Dallas and Cornelius 2015). In host–parasite systems, modularity has been interpreted as a reflection of ecological and evolutionary compartmentalisation shaped by host specificity, phylogenetic constraints or environmental factors (Krasnov et al. 2012, Costa et al. 2023). Therefore, the high modularity observed in these networks likely reflects biologically meaningful constraints shaping host use by parasitic copepods.

In the marine network, key fish species, such as *Mugil cephalus*, *Katsuwonus pelamis* and elasmobranchs like *Prionace glauca* and *Sphyrna mokarran* hosted a high number of copepod species and acted as structural hubs. Several of these species are currently classified as threatened or vulnerable by the IUCN (Dulvy et al. 2014, Froese and Pauly 2025), suggesting that their decline could lead to disproportionate losses of parasite diversity and erosion of network stability (Dallas and Cornelius 2015). Similarly, in freshwater systems, species such as *Micropterus salmoides* and *Micropterus dolomieu* served as important nodes. Although not currently threatened, their wide distribution and rich parasite fauna underscore their ecological significance.

Robustness simulations confirmed that targeted loss of highly connected host species causes steeper declines in copepod persistence, reinforcing their importance for maintaining network integrity. These findings highlight the role of certain fish species as keystone hosts in sustaining parasite diversity and network structure in aquatic ecosystems.

### Strengthening global copepod parasite datasets

The World of Copepods database provides an invaluable foundation for studying parasitic copepods globally. Nonetheless, parasitic copepods remain under-represented in macroecological studies despite their diversity and ecological significance. Advancing parasite macroecology will require: (1) sustained support for open-access databases; (2) inclusion of biotic and abiotic variables across spatiotemporal scales and (3) development of tools that streamline access to parasitic copepod data and support their integration into ecological workflows. Such efforts would empower investigations of host specificity, co-evolution and ecosystem resilience, directly supporting biodiversity conservation.

## Conclusions

This study provides a global-scale synthesis of host use and network structure in parasitic copepods infecting marine and freshwater fishes. The analyses reveal a predominant trend towards host specialisation, alongside the identification of generalist species with notable ecological and economic relevance. The modular structure of both marine and freshwater networks, coupled with their sensitivity to host loss, especially in marine systems where key elasmobranch hosts are threatened, emphasises the dependence of parasite diversity on host community stability. These findings highlight the value of host–parasite interaction data for understanding ecological complexity and support the integration of parasitic taxa into biodiversity assessments and conservation planning. Often overlooked, parasites can serve as sensitive indicators of ecosystem structure and resilience.
